# Share control of surgery robot master manipulator guiding tool along the standard path

**DOI:** 10.1002/rcs.1984

**Published:** 2019-02-19

**Authors:** Lukasz Fracczak, Mateusz Szaniewski, Leszek Podsedkowski

**Affiliations:** ^1^ Institute of Machine Tools and Production Engineering Lodz University of Technology Łódź Poland

**Keywords:** autonomous surgery robot, force control, minimally invasive surgery, share control, tool guidance

## Abstract

Recently, minimally invasive surgery (MIS) robotics enters the phase of autonomous operation. However, because of the high variability of the environment, conducting a fully autonomous surgery is still extremely difficult. This paper presents a share control system, the objective of which is to suggest the optimum path of tool guidance through the use of force on the master manipulator (hereinafter as master), meaning the surgeon's hand. Owing to this type of control, the surgeon has full control over the position of the tool the entire time and is supported by the system to better and faster guide the tool during surgery. The force should be felt by the surgeon but, simultaneously, must not hinder or impact the surgical process. Furthermore, the share control system presented in the paper can be turned on or off at any moment during surgery.

## 1 INTRODUCTION

Presently, minimally invasive surgery (MIS) robotics is entering a new stage of development, i.e. autonomous operation, Although there is still a long way to go until full autonomy is reached where the robot diagnoses and conducts surgeries on its own, scientists all over the world are working on procedures that will facilitate surgeons' work.^1^ These efforts mainly involve automation of movements that are repeatable^2^, ^3^ (eg, suturing), time‐consuming, or troublesome^4^, ^5^ (eg, tying knots). Correct design of the control architecture (ensuring the correct balance between the robot's autonomy and the surgeon's experience and intuition) is one of the biggest challenges associated with providing support for the surgeon.

The robot's operation autonomy can be divided into several groups: full autonomy, the robot performs some movements of the tool,^6^ cooperates with the surgeon,^7^ or performs the movements under the surgeon's supervision.^8^, ^9^ Share control over the tool's movements is one of the simplest solutions for the collaboration between the surgeon and the autonomous system. It is implemented by means of arbitrary weighting factors.^10^, ^11^ In this system, the values set by the surgeon are uploaded to the control system with weight “a” while the autonomous system affects the location of the tool with weight “1‐a.” Such a solution is effective when trainees learn the correct guidance of tools^10^, ^11^ during operation. In this case, the teaching surgeon has weight 1‐a, and the trainee has weight a. During the surgical operation, control division may bring a certain risk. It occurs because weighting factors are set before the procedure and can not be changed once it has started. A problem may arise in dangerous situations when a correction of the tool's path will have to be quick and by extensive values of displacement (when verifying the path, the surgeon must perform a movement of the master that will compensate for the movements generated by the autonomous system). Therefore, under this type of control, the necessity of reacting quickly (eg, in the case of artery or vein damage) may be extremely difficult, which puts the patient's health and life at a great risk.

In the case of control in which the autonomous system may perform some movements on its own, one needs to be certain that these movements are safe. Watanabe^6^ presents a system in which the surgeon controls two tools using only one master. In this situation, the autonomous system supervises one tool, while the surgeon is in charge of the second one. This system was used to perform a task during which the surgeon was intending to stitch a wound. At the time when the surgeon uses the tool to puncture the skin with the needle, the control system locates the point of puncture, moves the second arm independently to find the needle, and makes an attempt to grab it. After that, the control switches and the surgeon takes full control of the tool that grabbed the needle. Unfortunately, under real conditions, finding the point of puncture or grabbing the needle are activities that bring about certain problems. There are situations where the surgeon must make corrections in the stitching process. If the autonomous system is going to find and grab the needle during suturing corrections, then it may lead to incorrect suturing and subsequently postoperative complications.

Admittance control strategy characterized by time‐varying stiffness, damping, and inertia^12^ may serve as another example. The authors worked out a system where the control system was divided between stages of full autonomy and teleoperation. The “energy tank” method was applied in this system. Owing to that, the process of switching from a remote operation to a fully autonomous system is smooth. However, during an autonomous operation, the tool moves along the previously defined trajectory. Therefore, it moves in accordance with the predefined: trajectory, velocity, and pace of performing a certain activity, and with the possibility to adapt to varying external conditions. The systems where the surgeon supervises the work of the autonomous system provide much greater opportunities.^9^ In this case, the robot performs certain activities autonomously while the surgeon only corrects the trajectory. Such systems require the use of appropriate measurement sets, which monitor the location of tissues; and as a consequence, can adjust the trajectory of a tool's guidance to varying conditions (tissue location). Shademan^9^ describes such a system; however, it was carried out under laboratory conditions, and its implementation under normal surgical conditions is extremely difficult. Another method for the tissue position measurement is based on the small biocompatible near‐infrared fluorescent (NIRF) markers.^13^ This system measure the XYZ position without tissue orientations. The measured tissue position can be used for the correction of the tool's orientation and position. Besides complicated measurement systems, force sensors can also be used in medical robotics to analyze the working environment. A system examining whether there are any hard tissues in the patient's body.^7^, ^8^ The system presses the patient's body delicately and detects any hard tissues. During examinations, the system analyzes the shape and determines whether a given tissue is hard or soft. The results of palpation are visualized on the screen on an ongoing basis. Palpation was also used to compare different systems of human‐robot collaboration,^8^ for example: teleoperation, robot supervision (the surgeon gives instructions and the robots perform them), share control (the surgeon controls certain areas of autonomy), full autonomy, and traded control (the surgeon controls the robot and may give instructions to the robot to perform certain activities on its own). Based on various experiments conducted, the authors determined that a small level of autonomy could improve performance.

The force feedback control is another method that is used in the robotic assist surgery. There are a lot of methods^14^, ^15^, ^16^, ^17^, ^18^, ^19^ that differ each other with: measurement system, force implementation, etc. Generally, these types of controls are used to give to the surgeon information about the environment, eg, tissue stiffness, forces during the knot tying, and tissue grasping. This type of control is not suitable for the guidance tool along the paths. However, the system described in the paper can be merged with the force feedback systems as an another surgeon assistance feature of the surgery robot.

To sum up, no cardiac surgery robot is presently available that would significantly facilitate the work performed by surgeons and, simultaneously, would guarantee full safety for patients. Therefore, it is of the most importance to design such control architecture that will benefit from the advantages of autonomous systems (precision of movements, facilitation of repeatable and difficult movements) and teleoperation (the surgeon's experience, quick intervention in emergency situations, adaptation to varying conditions). Such a control system has been designed and is presented in this paper. The control system supports the surgeon during tool guidance. Therefore, the surgeon can operate the tool freely, without any restrictions. When help with tool guidance is required (tying knots, suturing, performing a difficult movement), operation assistance can be turned on. Then, the control system will only suggest the optimum trajectory of tool guidance but will not force the surgeon to rigidly keep to the predefined trajectory. Consequently the forces on the master are always orthogonal to the trajectory and do not initiate the motion along the path. This kind of control is named as the passive guiding tool.^20^ The main advantages of proposed system is to correctly guide the tool in the paths containing loops.

Few examples of share control systems can be found in the literature. The most similar one to described in this paper are presented by Shahin et al.^10^ and Marco de Baar.^21^ In work of Shahin,^10^ robot (slave manipulator) is an element subject to share control. In this system, master controlled by the surgeon has an influence on the master controlled by trainees, therefore both surgeon and trainees “feel” the differences between masters' positions. In this system, the position of the slave is an average value of both masters positions. In the presented share control system, guidance forces are on the master and are proportional to the distance between the actual position of the master and standard trajectory stored in the robot control system database; therefore, in our solution, the slave trajectory always coincides with the master trajectory.

In the system used by Marco de Baar,^21^ the master is guided along the control path. In this system, passive guiding tool with environmental interaction (insert the pipe in a hole) is used. The master is guided along the path with “look ahead guidance.” It is based on the estimation of the position of the tool in the future time (eg, 0,1 s), basing on the actual velocity vector and position. In the presented system, the tool is guided along the path basing on the current position of the slave and actual velocity of the master. It is done because the “ideal” path is defined relatively to the patient body in the robot coordinate system, but the surgeon controls the master position while he observes tool on the monitor.

## 2 METHODS

The general idea of the designed system is to treat the master as an object with shared force control—through drives (the autonomous part) and by the surgeon (manual part).^21^ Then, by controlling the position of the master, it is possible to affect the position of the surgical tool itself. Enabling the surgeon to correct the master's (surgeon's hand) position, hence the position of the tool as well is an extremely important aspect of master control. This is significant from the perspective of surgical tool guidance along a specific trajectory and the necessity to correct it because of varying external conditions, ie, the patient's body. Described above, surgery robot functionality is associated with the master's control along the implemented trajectory. It is applied using the action plan presented below:

1. Setting^4^, ^22^ and implementing standard trajectory into the robot control system.
2. Defining the space where the robot will follow the standard trajectory.^4^, ^23^
3. Determining the robot's position in relation to the implemented standard trajectory.
4. Determining the force value and direction on the master based on: the velocity on the standard trajectory and the tool's distance from the standard trajectory.
5. Setting force interactions on the master.
6. Determining the position of the master, i.e. determining how the surgeon moves the master (with set forces).
7. Transferring the set positions to the angular position in the slave manipulator's joints.
8. Movement of the robot (tool).
9. Repeating the activities from 3 to 8 until a specific procedure associated with the operation has been completed.

It is important that calculations at subsequent stages of the iteration are made in set positions of the slave manipulator's tool. Based on experiments conducted at the initial stage of the studies, such an approach to robot share control improves the smooth movement of the tool.

The points 1 and 2 of the action plan are described in others research works.^3^, ^4^, ^22^, ^23^ The standard path (SP) determination is one of the crucial problems. The surgical tool path depends on several conditions, and only in few situations the SP can be detected. This problem was discussed in details in Podsedkowski, Moll, Moll, and Fracczak's study.^3^

### 2.1 Determining the position of the robot's tool in relation to the standard trajectory

It is assumed that the tool is located outside the standard trajectory when share control is turned on (at the beginning of the task). Therefore, it is appropriate to find such a point on the SP that will be closest to the current position of the tool. In paper,^4^ the SP is described using a function. Then, the system is intended to calculate which of the points belonging to the function is closest to the tool's position. It all comes down to solve a system of equations, which is undoubtedly an advantage of the entire system. Meanwhile, such an approach is also associated with certain limitations regarding the shape of the function and keeping continuity in the case of more complex functions. Therefore, it is assumed that the standard trajectory is defined as a set of subsequently located points (positions which the tool needs to move along), whereas each point is defined based on a specific number of coordinates. With such an approach, the SP may have any shape desired (the manner of its registration is described in paper^3^ and later on in this article). Therefore, the path can be written down as

(1) $$\tau =\left\{X_{1},\ldots ,X_{n}\right\},$$

where X_*i*_ *i* = 1, …, *n* is the *i*th position of the tool, and the *i* index increases from 1 to *n* in the direction of planned movement, while X_*i*_ is the vector representing the space in which the position is registered (position can be determined in 2D, 3D, and 6D; the experiments presented in this article were conducted in 3D) and the speed of the cardiac surgery robot. Hence, it is possible to specify that $X_{i}=\left[x_{i},y_{i},z_{i},{\dot{x}}_{i},{\dot{y}}_{i},{\dot{z}}_{i}\right]=\left[p_{i},{\dot{p}}_{i}\;\right],$ where *p*_*i*_ describe position and ${\dot{p}}_{i}$ velocity of tool in *i*th point of the path. The entire path *τ* in relation to which the tool is to be guided will be referred to as the SP $\bar{\tau }$.

**Figure 1:**
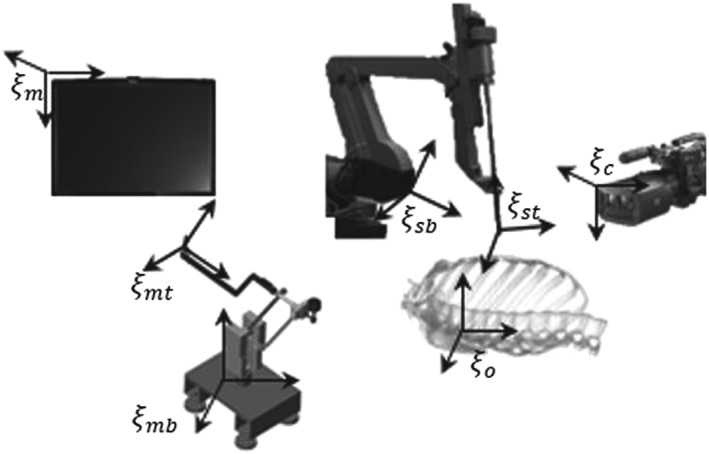
Main constituent parts of Robin Heart and designations of individual coordinate systems: ξ_mb_, the base of the master coordinate system (CS); ξ_mt_, the master effector CS; ξ_m_, the screen CS; ξ_sb_, the base of the robot CS; ξ_st_, the robot's effector CS; ξ_c_, the camera CS; and ξ_o_, the operation space CS

Control system studies were carried out on the Robin Heart surgery robot. This robot was developed in cooperation of Foundation of Cardiac Surgery Development (FCSD) and Lodz University of Technology.^24^, ^25^ This robot was successfully tested on the pigs; and nowadays, it is prepared for the commercial use as a camera‐holding robot. The architecture of Robin Heart's control has been created in a manner enabling the surgeon, who moves the master in a specific direction (eg, to the right), to see on the screen how the tool moves in the same direction (to the right). Additionally, it needs to be emphasized that any change of camera location in relation to the tool is possible and requires only a simple calibration procedure, which can be performed during operation.^23^ Therefore, it is possible to ascertain that the position of the SP in relation to the tool will be compatible, yet scaled up, to its position with reference to the master's effector. Owing to that, the direction of force interactions on the surgeon's hand, helping the surgeon to guide the tool along the SP, will be proportional to the deviations of the tool's position on the SP. All systems of coordinates linked with the control of Robin Heart 3 are presented in Figure 1, while their transformations were discussed in detail in paper.^23^

The position of the tool in relation to the path is determined based on the robot's current position *p*_*a*_ (in the camera system) and the current speed of the master ${\dot{p}}_{a}\;$(in the monitor system, which is identical to the camera system). Then, it is assumed that one of the points belonging to the SP is the set position $X_{d}=\left[p_{d},{\dot{p}}_{d}\;\right]$ (the point on SP with index *d* will be then threated as the desired position of tool). Therefore, it is appropriate to find such a point on the SP that will correspond with the current position of the tool the best. The entire path is sought during the first search for such a point. In subsequent iterations, the computational complexity of selecting a point is reduced, and only the position with an index from the following range is considered 𝑗 = *d* − 𝑘 to 𝑗 = *d* + 𝑘 where 2𝑘 + 1 is an arbitrarily assumed number of positions, and *d* corresponds to the last set position of X_𝑑_. Furthermore, the following conditions must be fulfilled: *j* > 1 and *j* < *n*. If these conditions are not met, the closest value *j* that already fulfills the conditions is accepted.

In order to select an appropriate point, each point on the SP is assigned the similarity parameter V_j_, which informs to what extent the given point fits the current position of the tool in terms of location. This can be written down as

(2) $$V_{j}^{\prime }=\left|p_{j}-p_{a}\right|\;\text{for}\;j=1,\ldots ,n,$$

(3) $$V_{j}=\frac{1}{V_{j}^{\prime }}\;\text{for}\;V_{j}^{\prime }\neq 0.$$

The point of which the similarity parameter will have the highest value will be the X_d_ point on the SP selected using this method. In order to ensure a smoother and, hence, more stable change of the current *d* index of the set position (X_d_), it is assumed the index *d’* corresponds to the geometric center of a figure created under the function (𝑗). It is determined based on the following dependence:

(4) $$d^{\prime }=\frac{\sum_{j=d-k}^{j=d+k}V\left(j\right)j}{\sum_{j=d-k}^{j=d+k}V\left(j\right)}$$

The index of the new set position *d* equals *d*', rounded to the nearest integer value.

Sometimes, the trajectory passes the same points several times, so adding velocity as an additional parameter to be compared in order to determine *p*_*d*_ corrects the quality of designation. However, several tests showed, when share control system is switched on and the master is not moving along the paths, velocity of master can disturb best point search process, therefore velocity is not included in the calculations at this point.

### 2.2 Determining the value and direction of the forces (selection matrices)

From the perspective of cardiac surgery robot's operation, turning on/off forces interactions along the set trajectory is crucial. It is unacceptable that the robot “pushes” the surgeon's hand, which would lead to excessive autonomy. It could result in improper tool guidance and damage of the patient's tissue. The cooperation between the robot and the surgeon should be in compliance with the following assumption: if the surgeon does not move the master at any time during operation, the robot must not make any movements that would continue the surgery. Otherwise, the robot would perform individual activities autonomously. Therefore, the control system's role involves mainly suggesting the optimum path for the tool by impacting the master with force in the direction perpendicular to the planned movement while the surgeon is still in charge of the pace of movement and guidance of the tool. This is possible by means of setting a temporary selection matrix (calculations and studies in the subsequent part of the paper are carried out on Cartesian linear coordinates (x, y, z) without an orientation analysis (φ, θ, *ψ*)).

Force interactions on the surgeon's hand will be proportional to the difference in the distance and velocity of the current position in relation to the SP. These forces are determined based on the following equations:

(5) $$F_{p}=K_{p}\left(p_{d}-p_{a}\right),$$

(6) $$F_{v}=K_{d}\left({\dot{p}}_{d}-{\dot{p}}_{a}\right),$$

(7) $$F=F_{p}+F_{v}.$$

Individual coefficients are appropriately equal: stiffness coefficient *K*_*p*_ = 0.1*k*_*p*_ and damping coefficient *K*_*D*_ = 0.01*k*_*p*_, whereas k_p_ is a scalar value. Szaniewski^26^ presents a value analysis of k_p_ parameter selection and its impact on the accuracy of tool guidance as well as the stability of operation of the entire system. Based on the results, the authors concluded that the accuracy of the movement tool along the SP increases with the increase of this coefficient. The value of k_p_ = 1 was used in the experiments and studies presented later on in this article.

**Figure 2:**
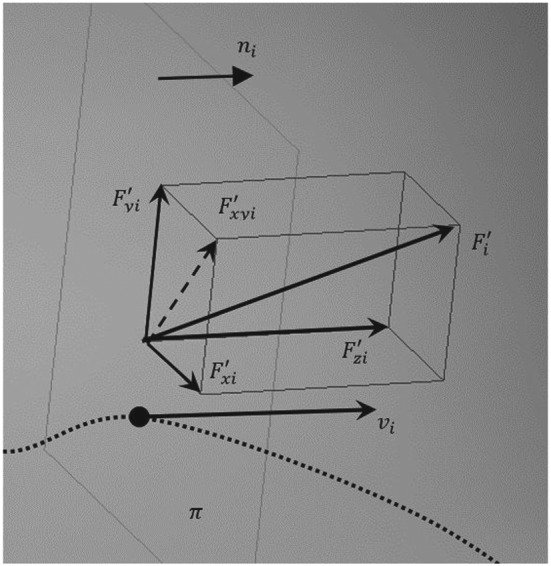
Force projection on the normal plane in relation to the velocity vector

Based on the set velocity ${\dot{p}}_{d}=v={\left[v_{x},v_{y},v_{z}\;\right]}^{T}$ corresponding to point *p* on the registered trajectory, it is possible to determine the plane perpendicular to vector *v* and described by the normal vector $n=\frac{v}{\left|v\right|}$ (Figure 2).

The $F_{v}^{\prime }$ component of vector *F*, being a projection of this vector on the velocity vector *v*, is determined by the following equation:

(8) $$F_{v}^{\prime }=n\left(n*F\right)=nn^{T}*F=\left[\begin{array}{ccc} n_{x}^{2} & n_{x}n_{y} & n_{x}n_{z}\\ n_{y}n_{x} & n_{y}^{2} & n_{y}n_{z}\\ n_{z}n_{x} & n_{z}n_{y} & n_{z}^{2} \end{array}\right]F=S_{\pi }F.$$

It was assumed, that the force vector *F*_*s*_ reacting on the operator's hand is always perpendicular to the velocity vector *v*_*i*_. It can be calculated using the following formula:

(9) $$F_{s}=F-F_{v}^{\prime }=F-S_{\pi }F=\left(I-S_{\pi }\right)F=\mathrm{SF},$$

where *S* = *I* − *S*_*π*_ represents the selection matrix. If the velocity *v* is perpendicular to force *F*, then *F*_*s*_ = *F*. If they are parallel, then *F*_*s*_ = 0. In addition, it was assumed that, if the

$${\dot{p}}_{d}=v=0\to S_{\pi }=0\to F_{s}=F.$$

Further on, the value of force interactions on the surgeon's hand need to be limited. Two factors should be taken into consideration in this aspect. The first coefficient involves the determination of the force value, which the surgeon considered perceptible, yet not interfering with the operation. The second limitation involves the maximum torque that can be generated by the masters's motors and which will not disturb the direction of force interactions.

First coefficient can be described in a simple way as

(10) $$F_{\lambda }=\left\{\begin{array}{c} F_{s}\text{if}\;\left|\vec{F_{s}}\right|\leq \lambda_{F}\\ \begin{array}{ccc} \frac{F_{s}\lambda_{F}}{\left|\vec{F_{s}}\right|} & \text{if} & \left|\vec{F_{s}}\right|>\lambda_{F} \end{array} \end{array}\right.$$

where *F*_*λ*_ is the vector of force affecting the surgeon's hand, and *λ*_*F*_ is a scalar value of the maximum force of interaction on the surgeon's hand (this value is assumed by the surgeon before the operation, in our experiments it was 7 N).

The second condition is set based on the values of current transferred to the motors, which are calculated as

(11) $$I\left(q\right)=K_{I}J^{T}\left(q\right)*F_{\lambda },\;$$

where *K*_*I*_ is a diagonal matrix of current amplification for individual motors and *J*(*q*) is the master's Jacobian matrix for joint variables vector *q*.

Current limitation is introduced as

(12) $$I\left(q\right)<\lambda_{I},$$

where *λ*_*I*_ is the vector of current limitations of the motors on individual articulated joints of the master. This vector is selected to ensure that the driving torque of the motors does not bring about changes in the forces generated on the master's handle. It needs to be noted that individual drive units should not be too weak, it should be provided during the selecting or designing the master stage. Otherwise, it may lead to a situation where it will not be possible to meet the stated inequality.

Owing to such an approach to the issue of force generated on the surgeon's hand, one may be certain that current limitations of the motors and the master's kinematics will not disturb the value and direction of force interactions.

It is also worth to mention that, if *λ*_*F*_ is sufficiently small, then the control will not limit the value of forces set because of current limitations.

**Figure 3:**
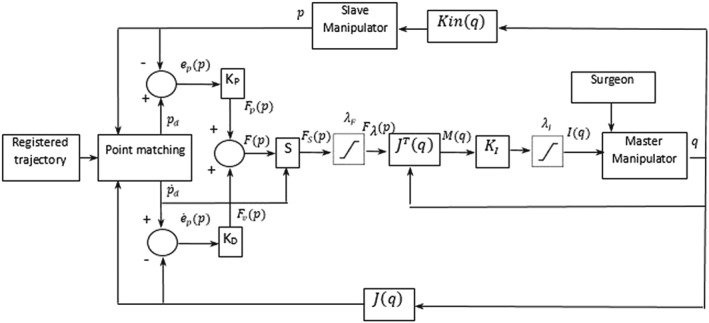
Schematic diagram of Robin Heart's control system

A block diagram of current‐assisted control, which is presented in Figure 3, has been worked out based on the deliberations presented above. This diagram is close to the scheme presented in papers.^26^, ^27^ On the basis of this diagram, it is possible to write down that the torque in the master's joints, M(q) are calculated according to the following formula:

$$M\left(q\right)=J^{T}\left(q\right)F_{s}\left(p\right)=$$

(13) $$=J^{T}\left(q\right)S\left({\dot{p}}_{d}\right)\left(K_{p}\left(p_{d}-p\right)+K_{d}\left({\dot{p}}_{d}-\dot{p}\right)\right),$$

where *J*^*T*^(*q*)—master's Transpose Jacobian Matrix, $F_{s}\left(p\right)=S\left({\dot{p}}_{d}\right)F\left(p\right)$ —selected force transferred perpendicular to the set velocity, and $S\left({\dot{p}}_{d}\right)$—selection matrix.

As is described above, the force generated on the master of the robot will always be perpendicular to the velocity between points on the SP, and the surgeon can guide tool because of his experiences. In the share control system described by Shahin,^10^ the forces are always generated in each direction and the values of the forces depends on masters position of two surgeons. Therefore, the position of the tool depends on the experiences and intention of both surgeons. Consequently, this system is good for teaching the trainees, but not to provide the operations.

## 3 EXPERIMENT

### 3.1 Analysis of tool guidance accuracy

**Figure 4:**
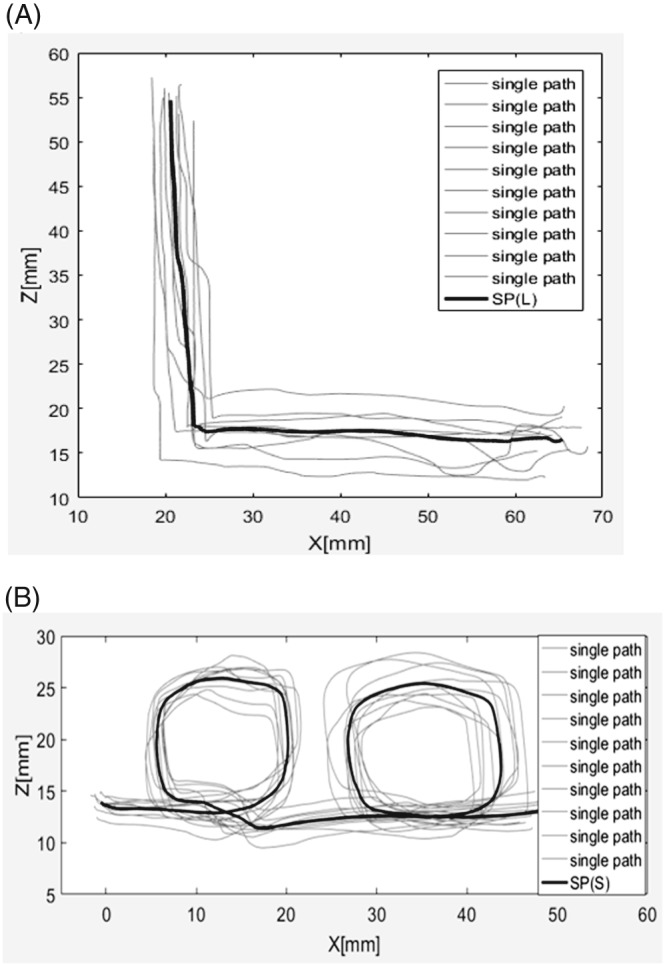
The recorded paths with calculated standard paths: A, L shape; B, S shape

The experiments were performed on the cardiac surgery robot—Robin Heart 3 (Figure 1).^22^, ^28^, ^29^ First of all, SPs were determined. Two paths shaped like the letter “L” and “Small Loops” (S) were drawn on a stiff piece of paper. Each shape was intended to verify how the control system determined points of the SP during operation in case of: sharp angles (L shape) and crossing and partially repeated sections of the SP (S). Then, the tool was moved along each shape 10 times without any assistance, ie, with master motors turned off. The system of coordinates associated with the piece of paper was defined each time based on the procedure presented in paper.^3^, ^26^ Such data were filtered using the finite impulse response (FIR) algorithm^30^ and then average paths were determined for each shape using the method described in paper^31^ (standard shapes will be hereinafter referred to as Standard Path “L” shape SP[L] and Standard Path Small Loops shape SP[S]). The results are presented in Figure 4.

Next, the SPs were implemented into the control system of Robin Heart 3. Then, the tool was guided 10 times along each SP with share control switched on. The time and position of the tool were measured during the experiment.

The following nomenclature is used in the further part of the article:

$\bar{\tau }$ ‐ the average path calculated based on the registered paths without share control. This path become the SP in the experiments with share control (SP[L] and SP[S] respectively).

$\sigma \left(\bar{\tau }\right)$ ‐ the standard deviation (SD) between registered paths.

without share control and $\bar{\tau .}$

$\overset{̿}{\tau }$ ‐ the average path calculated based on the registered paths with share control enabled. This path is used only for calculation the Standard Deviation of the paths with share control enabled.

$\sigma \left(\overset{̿}{\tau }\right)$ ‐ the SD between registered paths with share control enabled and $\overset{̿}{\tau }$

$\sigma \left(\bar{\tau },\overset{̿}{\tau }\right)$ – the SD between $\bar{\tau }$ and $\overset{̿}{\tau }$

$\bar{t}$ ‐ the average time of tool movement along the SP

W – the set of paths registered in the case of operation with share control disabled

SC – the set of paths registered in the case of operation with share control enabled

Designation example: W(L)—set of L‐shaped paths, SC(S) —set of Small Loops paths with share control enabled.

**Figure 5:**
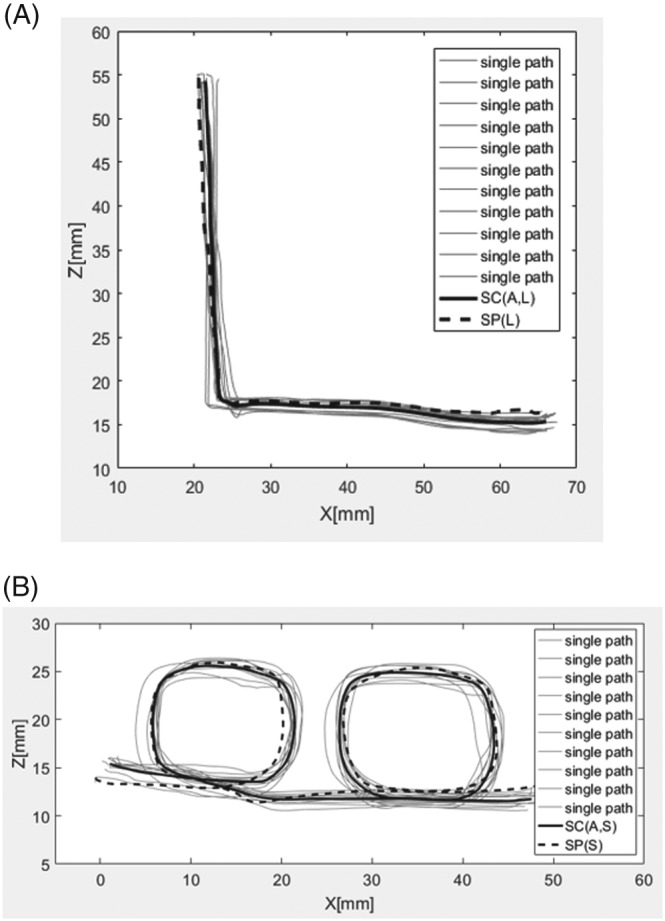
Paths indicating tool guidance with enabled share control: A, L shape; B, S shape; SC(A,L) is the $\overset{̿}{\tau }$ of the L shape, SC(A,S) is the $\overset{̿}{\tau }$ of the S shape

The results of the calculations are presented in Table 1, while the paths registered during tool guidance with force assistance (share control) are presented in Figure 5. This figure presents diagrams of the tool's position based on the coordinates Z and X. Meanwhile, all the SD calculations were performed in a 12‐element state space (location, orientation, and velocities versor, the orientation is multiplied by R coefficient, velocity versor is multiplied by T coefficient). This type of analysis is presented in paper.^32^ Individual values of σ (SD) were calculated using the following equation

(14) $$\sigma \left(\bar{\tau }\right)=\sqrt{\frac{1}{m}\sum_{k=1}^{m}\frac{1}{n_{k}}\sum_{i=1}^{n_{k}}\delta {\left(x_{i}^{k},\bar{\tau }\right)}^{2}},$$

**Table 1:** The average distances values and average time

Variable	Units	W(L)	SC(L)	W(S)	SC(S)
$\sigma \left(\bar{\tau }\right)$	mm	3.3	1.8	2.4	1.9
$\sigma \left(\overset{̿}{\tau }\right)$	mm	X	1.4	X	1.3
$\sigma \left(\bar{\tau },\overset{̿}{\tau }\right)$	mm	X	0.8	X	1.3
$\bar{t}$	s	12.7	8.5	19.3	14.1

where *k* is the number of the path, the points of which are currently compared with the average path; *m* is the number of paths, *n*_k_ is the last point of the *k‐th* path; and $\delta \left(x_{i}^{k},\bar{\tau }\right)$ is the distance between the *i*th point on the *k*th path ( $x_{i}^{k})$ and the average path $\bar{\tau }$.

In order to calculate the distance $\delta \left(x_{i}^{k},\bar{\tau }\right)$, the points ware matched using the dynamic time warping (DTW) method^33^ for all average paths, and distances were calculated using the following equation:

(15) $$\delta \left(x_{i}^{k},\bar{\tau }\right)=\left|x_{i}^{k}-x_{j}^{\tau }\right|={\left({\left(x_{i}^{k}-x_{j}^{\tau }\right)}^{T}W_{x}^{2}\left(x_{i}^{k}-x_{j}^{\tau }\right)\right)}^{\frac{1}{2}},$$

where ***x***_*i*_, ***x***_*j*_ are a pair of points that belong to the two different paths that are compared (in this example, $x_{i}^{k}$ lay on one of the simple paths and (*k‐th*) and $x_{j}^{\tau }$ lay on the average path),

*W*_*x*_ =  *diag* (1, 1, 1, *R*, *R*, *R*, *T*, *T*, *T*, *RT*, *RT*, *R*), is a diagonal matrix of the weight *w*_*i*_. *T* is the value according to which the velocities are calculated with reference to position values (in the experiment *T* = 0.07).

Based on the results presented in Table 1, it can be concluded that the σ parameters have a lower value when the tool is guided with share control, which can also be observed by comparing Figures 4 and 5. Furthermore, based on the results presented herein, it is evident that introduction of share control system increases the accuracy of tool guidance along the set trajectory ( $\sigma \left(\bar{\tau }\right)$ has a higher value for the paths registered with disabled share control for both shapes). It is significant that tool guidance with enabled share control was approximately 30% faster than without share control.

### 3.2 Analysis of the functional parameters of tool guidance

In this section, two experiments showing the functionality and stability of the system are presented.

The first study was intended to verify whether the algorithm designed actually limited the force interactions on the surgeon's hand. For this purpose, an experiment was planned, which involved moving the tool away from the SP in two different directions for the standard L path. The entire experiment was composed of the following stages:

1. Turning force control on.
2. Moving the tool away from the SP—this movement took place while the tool was moving along the path in order to visualize the increment of force and tool's distance from the path.
3. Moving tools as close as possible to the SP.
4. Change in movement direction.
5. Pull tools away from the SP

**Figure 6:**
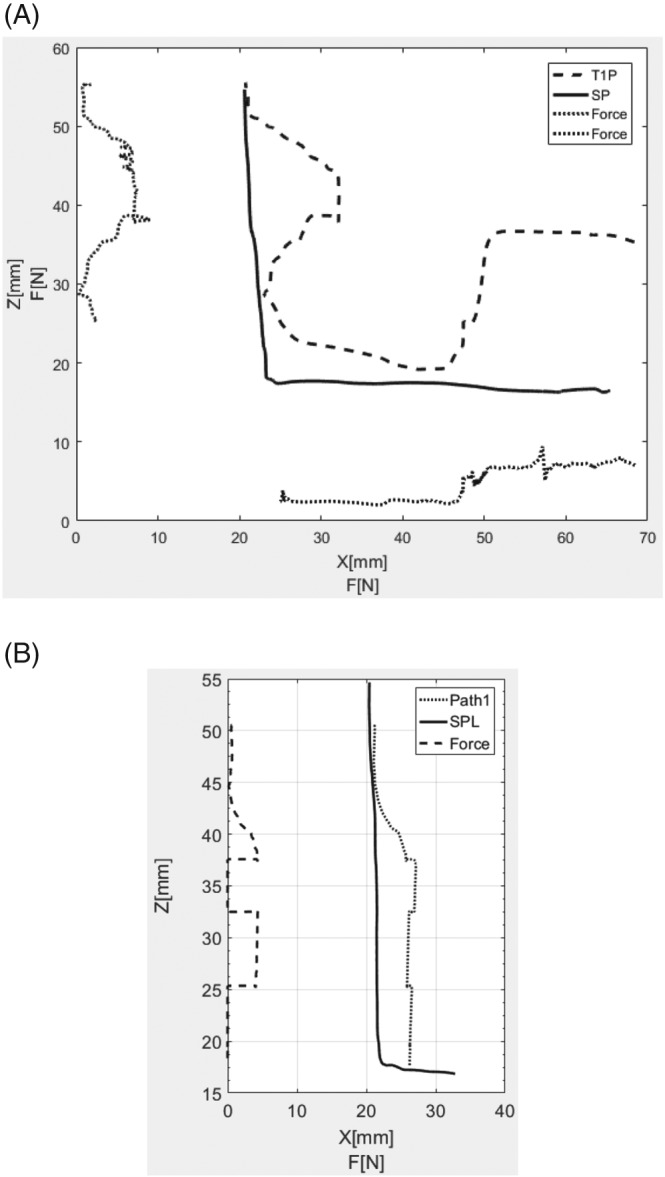
Force interactions and tool guidance in relation to the standard path: A, force limitation test; B, enabled and disabled force assistance test

This experiment was conducted with enabled assistance, at the same time, the trajectory and the values of the forces generated by the control system on the master were registered. The results of the experiment are presented in Figure 6A.

The forces presented in Figure 6 are calculated using the following equation:

(16) $$F=\sqrt{F_{x}^{2}+F_{y}^{2}+F_{z}^{2}}.$$

The figure also presents the SP used in share control algorithm and the real path of the tool motion (T1P) (Figure 6A). As is presented on Figure 6A, when the operator moves the tool away from the SP, the forces increased. In the about 48 mm (Z axis), the force stopped increasing (7 N value) despite the fact that the operator continued to move the tool away from the SP. Next, when the operator moves the master closer to the SP, the forces started to decrease when the distance between tool and SP was close enough. The analogous situation is presented when tool is guided along the X‐axis. This experiment shows that the force increases, while moving the tool away from the SP, it corresponds with theoretical assumptions.

Another experiment involved a verification whether force assistance could be enabled and disabled at any moment during operation. In order to do that, an experiment was planned, which involved guiding the tool along an L‐shaped vertical line. During guidance, the tool was moved away from the SP (with motor assistance enabled) and then tool guidance assistance was interchangeably disabled (in about 38 and 25 mm Z‐axis position) and enabled (in about 33 Z‐axis position) during the movement. The result of the experiment is presented in Figure 6B, where there are: the force of interaction on the master (force), the standard path (SPL), and the path along which the tool was guided (Path 1). It is proved that tool guidance assistance can be enabled and disabled at any moment during operation. It is important that the control system correctly defines the nearest point on the SP in relation to the tool's current position, which can be seen based on the small changes in the tool's position, which are perpendicular to the SP and were created at the time of enabling or disabling tool guidance assistance. Nevertheless, these changes are rather insignificant (approximately 1‐2 mm) and can be compensated by the application of the algorithm presented in paper^34^ or by the application of dynamically changing weights in conjunction with fuzzy logic systems.^35^

## 4 SAFETY AND STABILITY ANALYSIS

One of the medicine aspects is not to harm the patient. The same principle is actual in case of medical robots. And it was also the case while working on this surgeon assistance algorithm. It's done by:

1. removing the possibility of autonomous work—the robot does not move its tool by the path on its own, which is being achieved by implementing the selection matrix S;
2. adding forces restricting surgeon's hand movement to a level that can be easily overcome so he can guide the tool the way he wants to;
3. adding of dumping and stiffness coefficients that stabilize the motion.

An additional safety procedure will be implemented in final application. It will be based on the verification if the surgeons is holding master and is observing operation field.

The safety level using share control increases as the system prevents uncontrollable and pattern inconsistent motions to appear. The plan is that in final application for repeatable motions,^3^ an experienced surgeon will determine a method of unequivocal selection of coordinate system of given surgical procedure and will record this procedure several times. This repeats will be averaged^32^ to serve as SP. A great number of SP will serve as a database to be used by the robot's operator. Additional paths for such database can be obtained directly during the operation. In such case, the data of the tool's positions should be recorded; and next (after operation), the necessary data will be extracted for using them in future operations. There is also a possibility to define SP directly by the operator according to his experience and preferences. It can be done by using phantom for example.

Tool guiding system can be used for any shapes and lengths of SP, which makes this method universal. It needs to be pointed that enabling recording of SP directly from the robot enables exact guidance of tool during operation. Nevertheless, during operation, tissues can be slightly displaced, which makes it necessary to adjust the tool. Therefore, for tool slightly displaced from SP, the guiding forces will be small (Figure 6) so the surgeon could easily correct the path to perform operation correctly. In case of emergency, the system can be switched off completely in any moment in order to extend overall safety even further.

The stability of the system cannot be proved theoretically as one of the system's elements is a human being. The damping and compliance of human hand differs for each individual and is being determined by muscles tension. This problem is widely discussed in Szaniewski, Podsędkowski's study.^26^ Basing on the presented results, stiffness K_p_ and damping K_d_ coefficients were selected. The correctness of this selection has also been confirmed during tests presented in previous sections. Figure 6B presents systems response for step input. When tool guidance is turned on (ca. at 35 mm in Z‐axis), than there is no overshoots and oscillations on master position diagram (Figure 6B). Force stabilization time is ca. equal 0.3 seconds, therefore it can be stated that system not contacting tissues is stable. It needs to be stated that system being stable while lacking contact with the tissues will be remaining its stability after such contact occurs. It's because of fact that contact with tissues generates additional stiffness and damping of the system, which results in greater stability.

## 5 CONCLUSION

The method of force interactions presented in this article is destined to support the surgeon while performing repetitive movements or when the movements are difficult to perform. Several aspects are of significance in the presented method. One of them is that the surgeon can define, independently, the trajectories along which the surgery will be conducted. This way, the surgeon is given an opportunity to implement any path desired to the control system, ie, during suturing, tying knots, or in the case of difficult movements that need to be made with extreme precision during surgery. Additionally, the surgeon may, at any time during the surgery, enable or disable tool guidance assistance without the necessity of discontinuing operation. It is sometimes decisive when external conditions change—ie, tissue rupture, sudden bleeding, etc. The very manner of force interactions implementation is an important aspect. Force interactions are proportional to the tool's distance from the nearest point on the SP. Obviously, maximum force is limited to such an extent that it is perceptible on the master's handle but still not too high to hinder the operation of the cardiac surgery robot. Such an approach allows the surgeon—with share control enabled—to still be able to correct the path of the tool without using excessive forces.

To sum up, implementation of such a control system gives the surgeon full control over the position of the cardiac surgery robot with simultaneous movement assistance ensuring that the trajectory of the tool is in compliance with the previously defined model. The results of the studies presented herein can also be applied to train surgeons using a training stimulator with implemented share control, which would teach surgeons' correct guidance of the tool also in the case of surgery without a cardiac surgery robot.
